# Perceptions and Acceptability of Short Message Services Technology to Improve Treatment Adherence amongst Tuberculosis Patients in Peru: A Focus Group Study

**DOI:** 10.1371/journal.pone.0095770

**Published:** 2014-05-14

**Authors:** Sandra Albino, Karen M. Tabb, David Requena, Miguel Egoavil, Maria F. Pineros-Leano, Joseph R. Zunt, Patricia J. García

**Affiliations:** 1 Unit of Epidemiology, STD and HIV School of Public Health and Administration, Universidad Peruana Cayetano Heredia, Lima, Peru; 2 School of Social Work, University of Illinois at Urbana-Champaign, Urbana, Illinois, United States of America; 3 Center for Latin American and Caribbean Studies, University of Illinois at Urbana-Champaign, Urbana, Illinois, United States of America; 4 Departments of Neurology Medicine and Epidemiology, University of Washington School of Medicine, Seattle, Washington, United States of America; 5 Departments of Global Health and Neurology, University of Washington School of Medicine, Seattle, Washington, United States of America; Public Health Agency of Barcelona, Spain

## Abstract

**Background:**

Tuberculosis (TB) is global health concern and a leading infectious cause of mortality. Reversing TB incidence and disease-related mortality is a major global health priority. Infectious disease mortality is directly linked to failure to adhere to treatments. Using technology to send reminders by short message services have been shown to improve treatment adherence. However, few studies have examined tuberculosis patient perceptions and attitudes towards using SMS technology to increase treatment adherence. In this study, we sought to investigate perceptions related to feasibility and acceptability of using text messaging to improve treatment adherence among adults who were receiving treatment for TB in Callao, Peru.

**Methods:**

We conducted focus group qualitative interviews with current TB positive and non-contagious participants to understand the attitudes, perceptions, and feasibility of using short message service (SMS) reminders to improve TB treatment adherence. Subjects receiving care through the National TB Program were recruited through public health centers in Ventanilla, Callao, Peru. In four focus groups, we interviewed 16 patients. All interviews were recorded and transcribed verbatim. Thematic network analysis and codebook techniques were used to analyze data.

**Results:**

Three major themes emerged from the data: limits on *health literacy and information* posed challenges to successful TB treatment adherence, *treatment motivation* at times facilitated adherence to TB treatment, and *acceptability of SMS* including positive perceptions of SMS to improve TB treatment adherence. The majority of patients shared considerations about how to effectively and confidentially administer an SMS intervention with TB positive participants.

**Conclusion:**

The overall perceptions of the use of SMS were positive and indicated that SMS technology may be an efficient way to transmit motivational texts on treatment, health education information, and simple reminders to increase treatment adherence for low-income TB patients living in Peru.

## Background

Tuberculosis (TB) is a global health concern and a leading cause of mortality in developing countries. While rates of TB are slowly declining worldwide, an estimated 8.6 million cases of TB were registered in 2012 [1]. Reversing the incidence of diseases, including TB, is a major target of the United Nations Millennium Development Goals and requires innovative strategies to combat diseases. The incidence of TB is greater in developing low- and middle income countries [2]. Peru is a developing country with a high, yet declining, rate of TB cases. In order to address the high incidence of TB, the Peruvian government has adopted the strategy launched by the World Health Organization (WHO) called DOTS (Directly Observed Therapy – Short Course). This strategy requires patients with TB to establish and adhere to a rigid 6-month daily administered treatment regimen. Although this strategy has achieved success rates of more than 85% in TB patients, it has not been effective at achieving WHO targets and TB remains a major public health concern in Peru [3]. DOTS treatment adherence is critical, and the consequences of treatment failure are of great concern. Patients who do not complete treatment are more likely to relapse or die and are also vulnerable to the development of multi-drug-resistant tuberculosis (MDR-TB), which can then be transmitted to new cases. Given the importance of treatment success, it is necessary to support the DOTS strategy with the addition of innovative components to increase program effectiveness in treating infectious disease among Peruvians [4], [5].

The main barrier to effectively treating infectious diseases is failure to adhere to treatment [5]–[7]. A systematic review of qualitative studies indicated that the main reasons patients did not adhere to TB treatment were difficulty accessing healthcare, stopping treatment because the patient felt well, financial burden, and lack of understanding regarding treatment [6]. For some, inappropriate health-seeking behaviors such as delaying or disrupting DOTS treatment is related to traditional healing beliefs in low-resource communities [8]. Other major reasons for poor compliance are prolonged treatment duration, lack of transportation, forgetfulness [9]–[11], and limited knowledge about disease [12], [13]. Finally, one critical barrier to treatment is that patients miss appointments at health centers, which sometimes means they stop treatment prematurely. Given these barriers, it is necessary to support the DOTS strategy with the addition of innovative components to increase program adherence in Peru.

Use of information and communication technologies, such as sending reminders through short message services (SMS), can bring potential innovations to address barriers to treatment adherence. Mobile phone penetration is increasing in developing countries, thus using phones for healthcare treatment (mHealth) has potential to reach more people than traditional forms of disease control [14], [15]. The use of SMS messages to support healthcare has been tested in several low-income countries and resulted in a significant improvement in treatment adherence for patients with asthma [16], diabetes [17], HIV [9], [18]–[20], and TB [5], [21], [22]. The effectiveness of SMS reminders to improve TB treatment adherence yields inconsistent evidence, and this limitation is attributed to small samples and lack of appropriate controls [23]. Nevertheless, a recent systematic review finds that while mobile phones can deliver health messaging to patients directly, there remains a paucity of research on the effectiveness of SMS to improve TB treatment [23]. In a qualitative follow up of a pilot intervention of 30 TB patients with mobile phones, daily SMS reminders were found to have a positive impact and participants found the reminders encouraging [21]. Despite recent studies, little is known regarding the successful implementation of SMS to improve treatment adherence with TB patients in Peru. Even less is known about whether the patients will feel comfortable receiving SMS reminders about DOTS to improve TB treatment adherence. Thus, we decided to examine the attitudes and perceptions of current TB patients from Peru on the use of SMS as a tool to improve treatment adherence in the national DOTS program.

### Study Setting

Peru has one of the highest rates of TB incidence (106 per 100,000 habitants) in the Americas [1]. In 2007, there were 37,015 registered cases of TB in Peru [3]. In 2010, TB incidence in the Callao region was 121 per 100,000 habitants, higher than the national rate [24]. At the same time, the number of mobile phone lines in Peru, a country with 29 million people, has increased from 2.9 million to 28.9 million operational lines in 10 years [25]. Therefore, an assumption exists that the average Peruvian might have access to a mobile phone. Presently, even the simplest phones are able to receive SMS, providing an efficient and cost-effective way to communicate with wide ranging types of mobile phones in Peru [25].Accordingly, there is an opportunity to use the devices as a vehicle to transmit health-related information to low-income TB patients living in Peru.

## Methods

### Ethics Statement

All study protocol and instruments were approved by the Institutional Review Boards of the Universidad Peruana Cayetano Heredia, the University of Washington, and the Callao Directorate of Health. Participants provided informed verbal consent before the start of each focus group after facilitators read a consent form to the group. Verbal consent was chosen over written consent to avoid potential issues with literacy and because personal information such as names or other identifiers was not recorded. All participants were able to stay in the focus group provided verbal consent was obtained, and they were free to leave the focus group if they did not consent. The verbal consent was audio recorded, without names or personal information. This procedure was also approved by the Institutional Review Boards.

### Sample

We recruited a convenience sample of TB patients currently in treatment at health clinics in the region of Callao who had completed at least 2 weeks of treatment prior to consenting to the study. We identified four health centers in Callao (all from the Ventanilla Health District) based on approval from the local health board and on the high prevalence of TB in these specific health centers. Nurses working at the TB program in each of these centers were invited to attend two training sessions on the objectives and methods of the study, ethical principles of research, recruitment, and obtaining informed consent. After the training, six nurses volunteered to recruit participants for the focus groups. All participation from nurse recruiters was voluntary and no compensation was received. Participants were approached by the nurses at the four public health centers and were given an introduction to the study. If interested, they were invited to participate in the focus groups and given the time and location for the interviews. On the day of the interview, the study description was provided by the investigator and interview facilitator. All participants provided informed consent and permission to audio record the interviews. Participants received monetary remuneration equivalent to $4 USD to cover the cost of transportation to the focus group interviews.

### Data collection

**Figure 1:**
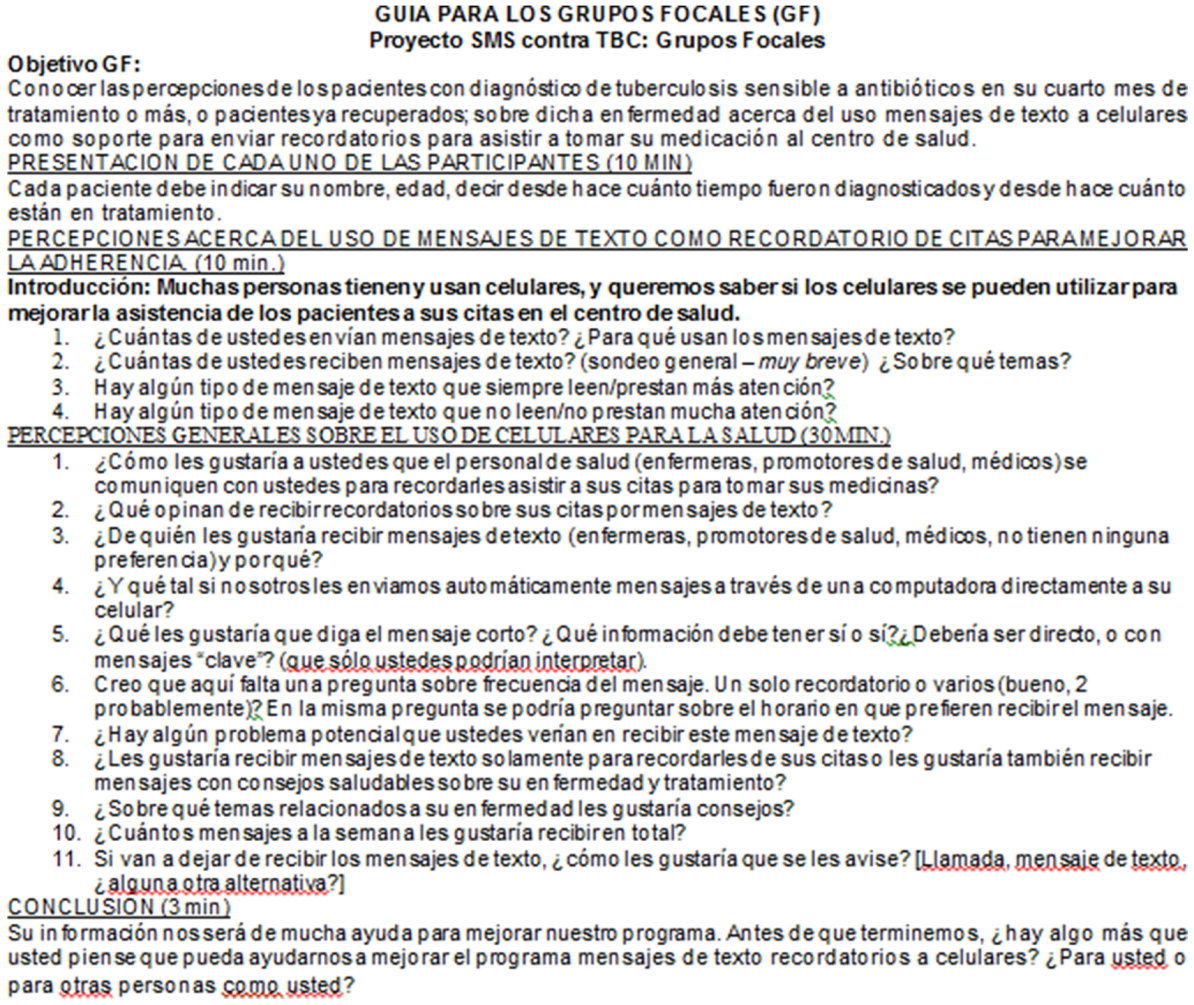
Focus Group Guide.

During August and September 2012, we conducted four focus groups with TB patients. At the start of each interview, we collected socio-demographic data including age, sex, marital status, occupation, region of origin, language, and ethnicity from each participant, which was linked to pseudonyms for identification. Participants' complete names and personal data were not recorded during the interviews. We used a semi-structured interview guide for all four focus groups (see Figure 1). Focus group interviews lasted an average of 50 minutes. A trained, experienced, native-Peruvian, Spanish-speaking facilitator conducted the focus groups with a secondary facilitator as note taker (note takers included following authors: M.E., S.A., D.R.). In the verbal informed consent process, all participants had provided permission to audio record the interviews. The focus groups were audio recorded and transcribed verbatim in Spanish. All translations from Spanish to English were conducted by the investigators (including the following authors S.A., M.P., K.T.).

### Data Analysis

We used thematic network analysis and a codebook technique to conduct qualitative analysis of the transcripts for four focus groups. Three raters (raters include the following authors S.A., K.T., D.R.) read the transcripts and developed a coding framework. All transcripts were read individually and discussed in detail to obtain inter-rater reliability. Using a codebook, four dozen initial codes were created and reduced to themes during regular inter-rater data analysis meetings. Themes were reapplied to the transcripts, and two themes were taken out of the data summary. In the final analysis, we found three descriptive themes to summarize the data.

## Results

The focus group interviews included 16 non-contagious tuberculosis patients from diverse backgrounds. The majority, 93% of the sample, preferred to speak Spanish instead of Quechua. The median age of participants was 38 years (interquartile range, 2952 years). Place of origin varied across the sample, with 37.5% from the Andes Mountains, 31.25% from the city of Lima, 18.75% from surrounding Lima, and 12.5% from the Amazon jungle. Half of the sample participants were married or cohabitating, and half were separated or single. The sample varied in educational attainment: 37.5% had completed secondary school, 31.25% had completed primary school, 18.75% had not completed primary school, 6.25% had completed a technical program, and 6.25% had completed superior school. All of the participants but one lived with other people in the same home, ranging from 2–8 people (mean 4.73, s.d. 2.05).

We used a thematic network approach and codebook to analyze all data and found three organizing themes to gain an understanding of the global theme “SMS to improve TB treatment adherence.” The three organizing themes were: *health literacy and health information* challenges to treatment adherence, facilitators and *motivators for treatment adherence*, and perceptions on the *acceptability of SMS for health*. Themes and illustrative quotes are presented in the following sections.

### Health Literacy and Health Information

Health literacy and health information emerged as salient themes from all four focus groups. Initial codes such as beliefs and lack of information are captured under this single theme. Many patients shared that they did not receive the right information or were not able to retain the information given to them. Accordingly, participants expressed that they did not fully know the importance of the treatment.

*“…when you just enter the program and the meetings, or the interviews, talks; let's say that in that moment when you receive the diagnosis of TB, you forget everything because your mind is not paying attention…”*

To reduce health literacy barriers, participants were asked for their views on an SMS system that could remind them about their appointments and offer motivational support and informative information about TB. In addition to being open to receiving individual health information via SMS, the participants mentioned they would like to receive text messages reminding them to take their acquaintances for screening. They responded they would like to receive these kinds of messages on their personal mobile phones or on family mobile phones.

*“…patients do not receive the right information, don't know the importance of taking the pills…then if you sent a text message with that information, it would make you more aware…”*

Participants reported that not understanding the information was a challenge for treatment adherence and also for describing the importance of screening to others. Patients indicated that even though they received information about the correct protocol and suggestions for screening of their contacts, they did not understand the information well enough to tell others. Many participants felt that information was not explained well to them. At the same time, many patients shared that they had attempted to take their relatives to the health center for tuberculosis screening, but some of them did not go because they were not aware about the real possibility of developing the disease. Patients also indicated that family members held a fear of being diagnosed, they did not know the importance of early diagnosis, they did not think they were going to be infected, they did not have symptoms themselves, or they feared medication. These shared descriptions provide insight into the community norms and perceptions of screening for TB.

### Motivation for Treatment Adherence

There were a multitude of opinions regarding the facilitators of TB treatment adherence. For some participants, minimizing the stigma of the disease was a successful motivator to continue DOTS treatment and visit the health clinics. As described by one participant, a missed appointment could potentially result in a home visit from a health worker and this might draw unwanted attention from neighbors in the community:

*“For example, some mornings I feel unmotivated, and chilly…and I know they [health center personnel] will call me or come to my house, and you know the nosy people [laughing], because everything goes mouth to mouth, so in order to avoid that I prefer to come see the doctor.”*

Participants were asked about motivators to continue treatment, and many expressed that they were aware of the seriousness of TB and that the fear of death were a powerful motivators. Thus the participants were taking the steps to be healthy and become cured. The theme of motivation also included the following codes: trying to avoid an early death, to go on with life, or steadfast because of characteristics of their personality.

Family emerged as a salient subtheme as many participants described family as a strong reason for continuing treatment. Key motivators included having family to financially support, trying to avoid infecting their families, being aware of the disease, and being able to care for their families. An example, of the importance of family relationship is described as follows:

*“Also to avoid being contagious to other people and our family that love us, for me it would be terrible to infect my family, my brothers. It's enough for me to have this, but I want to avoid that [being contagious], that is why I continue my treatment…”*

In summary, participants were motivated to become healthy either to support their families or to prevent their family members from contracting the disease.

### Acceptability of SMS to supplement TB treatment

The groups were asked if text messages could be used to improve health (mHealth). All participants expressed that they would like to receive health-related text messages. Many hoped such messages would encourage them to continue treatment and would inform them about diseases (including but not limited to TB). Participants suggested the messages could provide information on how to control TB and help them know more about TB. In addition to providing knowledge regarding prevention of TB, the messages could remind them to take their medication and attend appointments. The text messages were described as potential guides to keep patients updated on their disease status during treatment. Last, the text messages could be used to inform participants' family and friends about TB.

It was clear from all participants that the messages would need to be informative yet discrete in order to protect the patient from stigma in the community. One participant expressed apprehension about how others would perceive the text messages:

*“[In the event that]… somebody grabs my cellphone. Yes, I would feel bad, because then they will know that I have this disease. And everything will be different, they would try to keep me away from their children… [they might say] “don't let her get closer because she can infect you [with tuberculosis].” It's bad [that] there is a lot of discrimination. Especially when your kids play together, they say, “no [you cannot play because] they are kids from the neighbor, and they can infect you.”*

The participants were also asked about possible benefits the text messages could have. They responded that text messages were the cheapest, fastest, and best way to communicate, and also offered the possibility of storing relevant information from the text messages:

*“… it [SMS] doesn't have [to include] anything [potentially viewed as] negative. Because it [SMS] would not have names on it, so [it would be like] every [other] time you get many text messages from everything.”*

In order to assess possible negative effects, participants were asked about possible problems. They responded that discrimination could be an issue if somebody noticed or read the messages related to TB. Furthermore, participants expressed their preference for receiving messages without identification or names that could identify them as tuberculosis patients.

*“Maybe they are beside you, aware of it [tuberculosis], talking to you, but sometimes they don't want to understand, […] sometimes you feel bad, because even though you try to explain them how is it [tuberculosis], they don't want to understand, some people ignore you at their houses.”*

Despite these concerns, when asked how to manage possible stigma of the disease and potential negative aspects, participants stated they could handle it by deleting the messages after reading them. When asked about the specific message content they would find helpful, participants reported several motivational and reminder phrases and also asked for low budget and healthy cooking advice. They also declared they would like to receive congratulatory messages for every step or for continuing their treatment.

In terms of the frequency and timing of the reminders, the majority preferred the afternoon or evening 1 day before an appointment. Also, some proposed receiving reminders on the day of the appointment, a few hours before, or in the morning when they wake up. Finally, the majority of participants in our study expressed their dislike for receiving messages on Sundays.

## Discussion

We conducted focus groups with current TB patients to understand their views about treatment adherence and their perceptions regarding the use of mobile phone text messages as a way to improve treatment adherence. Participants identified barriers and facilitators to treatment adherence as well as discussed the use of SMS messages as an intervention to overcome this obstacle. We presented the findings in three distinct themes: health literacy and information, SMS acceptability, and motivation for treatment.

Health literacy needs and the desire for health information through SMS emerged as a theme in our focus groups. They expressed they would like to receive motivational and congratulatory messages, in addition to appointment information and reminders of their visits. These results are similar to past studies that also found participants would prefer motivational reminder messages [26]. Moreover, participants also expressed that they would prefer clear, confidential messages. Similar results were found by Lester and colleagues in 2006 and Curioso and colleagues in 2009, where one of the main issues related to the use of technology had to do with assuring confidentiality and not having messages that could disclose the health status of the individual [26], [27].

Family support and consciousness for the health of significant others emerged as key facilitators or motivators to improve treatment adherence for participants. These findings align with prior studies of treatment adherence in developing countries where perceptions and lack of knowledge regarding recovery from disease were related to failure to adhere to treatment [28], [29]. In our study, several participants shared that when they started to feel better they stopped DOTS treatment. This is an area where health education messages could prove beneficial, and participants agreed there were multiple potential benefits for using text messages as a tool to increase adherence to TB treatment.

Overall, participants offered insightful information related to using text messages as a reminder tool to increase adherence to TB treatment. Responses indicate that use of text messaging with patients from Callao, Peru, could potentially be well received, and participants provided valuable information as to what content should be included. Patient support for the use of SMS aligns with related studies on enhancing treatment adherence for other infectious diseases in developing countries. For example, Pop-Eleches and colleagues carried out a randomized control trial in Nyanza Province, Kenya, in which researchers sent daily, weekly, or no reminders to HIV/AIDS patients. Compared to patients who did not receive SMS reminders, weekly reminders increased treatment adherence by 13% to 16% [18]. In a similar study, Hoffman and colleagues indicated that participants felt Mobile Direct Observation of Treatment (MDOT) was a viable alternative to DOT (Direct Observation of Treatment), and patients preferred the MDOT service over DOT. Furthermore, Hoffman and colleagues found that SMS messaging helped patients feel less isolated and that patients valued the reminders and welcomed messages regarding health-related issues [4]. Further studies should focus on carrying out a pilot study to determine the feasibility and effectiveness of the use of text messages to address treatment adherence among TB patients in Peru.

There are several limitations to this study. First, we relied upon a convenience sample framework and thus our sample was limited to individuals compliant with treatment who were receiving care during a particular timeframe. It is not representative of all TB patients in Peru. Due to biosecurity issues, we recruited only patients who were actively on treatment and were sputum negative. Accordingly, we do not have information on the perceptions of SMS to improve TB treatment from patients who completed treatment successfully and patients who live outside of the Callao region. Second, our interview approach only included first-person respondents and individuals were asked to describe their own experiences. Culturally, the narratives and experiences of our participants were collective. The focus group often captured stories about family history or friends and/or treatment within the family household, including what they have heard or seen in their environments. In addition, the first-person responses came from participants who were of middle age, 50% of whom were married, thus social stigma may have been lower than in groups, such as younger married people. Last, our sample was limited to individuals currently under treatment, and a larger sample could have been obtained if retrospective accounts had been sought.

## Conclusions

Despite efforts to combat infectious diseases globally, the incidence of TB and barriers to treatment persist in low- and middle-income countries. Innovative and culturally appropriate strategies such as the use of SMS reminders have potential to increase infectious disease treatment adherence. Yet few studies have examined patient perspectives on the acceptability and feasibility of introducing SMS messaging to improve TB treatment adherence in Peru. The findings from this qualitative study show support for text messaging at the community and clinic level to try to improve treatment adherence in TB in Peru. Future studies are needed to pilot the use of an SMS intervention to improve treatment adherence for low-income tuberculosis patients living in developing countries such as Peru.
